# Combining information on multiple instrumental variables in Mendelian randomization: comparison of allele score and summarized data methods

**DOI:** 10.1002/sim.6835

**Published:** 2015-12-13

**Authors:** Stephen Burgess, Frank Dudbridge, Simon G. Thompson

**Affiliations:** ^1^ Department of Public Health and Primary CareUniversity of CambridgeCambridgeU.K.; ^2^ Department of Non‐communicable Disease EpidemiologyLondon School of Hygiene Tropical MedicineLondonU.K.

**Keywords:** Mendelian randomization, weak instruments, instrumental variables, causal inference, genetic variants, summarized data, aggregated data, allele score, genetic risk score

## Abstract

Mendelian randomization is the use of genetic instrumental variables to obtain causal inferences from observational data. Two recent developments for combining information on multiple uncorrelated instrumental variables (IVs) into a single causal estimate are as follows: (i) allele scores, in which individual‐level data on the IVs are aggregated into a univariate score, which is used as a single IV, and (ii) a summary statistic method, in which causal estimates calculated from each IV using summarized data are combined in an inverse‐variance weighted meta‐analysis. To avoid bias from weak instruments, unweighted and externally weighted allele scores have been recommended. Here, we propose equivalent approaches using summarized data and also provide extensions of the methods for use with correlated IVs. We investigate the impact of different choices of weights on the bias and precision of estimates in simulation studies. We show that allele score estimates can be reproduced using summarized data on genetic associations with the risk factor and the outcome. Estimates from the summary statistic method using external weights are biased towards the null when the weights are imprecisely estimated; in contrast, allele score estimates are unbiased. With equal or external weights, both methods provide appropriate tests of the null hypothesis of no causal effect even with large numbers of potentially weak instruments. We illustrate these methods using summarized data on the causal effect of low‐density lipoprotein cholesterol on coronary heart disease risk. It is shown that a more precise causal estimate can be obtained using multiple genetic variants from a single gene region, even if the variants are correlated. © 2015 The Authors. *Statistics in Medicine* published by John Wiley & Sons Ltd.

## 1 Introduction

An instrumental variable (IV) can be used to estimate the causal effect of a risk factor on an outcome from observational data 1, 2. A valid IV must be associated with the risk factor of interest but not associated with other factors on alternative causal pathways. This implies that it is not associated with any confounder of the risk factor–outcome association and that any causal pathway from the IV to the outcome passes through the risk factor 3. Much recent attention has been devoted to IV analysis in the context of Mendelian randomization, defined as the use of genetic variants as IVs 4, 5.

The causal effect of the risk factor on the outcome with a single IV can be estimated by dividing the coefficient from the regression of the outcome on the IV by the coefficient from the regression of the risk factor on the IV 6. This is known as the ratio of coefficients method. Alternatively, the same estimate can be obtained by first regressing the risk factor on the IV and then regressing the outcome on the fitted values of the risk factor from the first‐stage regression 7. This is known as the two‐stage least squares (2SLS) method. The 2SLS method can be extended for use with multiple IVs 8. As the number of IVs increases, overfitting in the first‐stage regression model leads to systematic finite‐sample bias in the causal estimate 9. This bias, known as weak instrument bias, acts in the direction of the confounded observational association between the risk factor and outcome 10. When there is a single IV, the median bias of the ratio (or 2SLS) method estimator is negligible for all but the weakest of IVs 11. A recent methodological development to exploit this fact is to aggregate multiple IVs into a single univariate score, and to use this score as a single IV rather than to use multiple IVs 12. In Mendelian randomization, this is known as an allele score, genetic risk score or gene score.

An alternative approach to combine information on multiple IVs is to use summarized data on the associations of genetic variants with risk factors and disease outcomes. These data are increasingly becoming available from large consortia, such as the Global Lipids Genetics Consortium (GLGC) for lipid fractions 13 and DIAGRAM for type 2 diabetes 14. Causal estimates can be obtained from these associations for a single genetic variant using the ratio method without the need for individual‐level data. Two methods for obtaining causal estimates from summarized data for multiple IVs have been proposed: a summary statistic method, in which the ratio estimates from each IV are combined in an inverse‐variance weighted meta‐analysis 15, 16, and a likelihood‐based method, in which the summarized data are modelled directly using a likelihood function 17, 18. The summary statistic method requires that the IVs are uncorrelated in their distributions (for genetic IVs, the variants are in linkage equilibrium).

In this paper, we review and extend the literature on IV estimation methods with summarized data, currently described in disparate sources. In Section 2, we lay out the assumptions made in this paper for the identification of causal effects. In Section 3, we demonstrate how an allele score estimate with a pre‐specified choice of weights can be reproduced using summarized data. We rederive the known result for uncorrelated IVs that the allele score and summary statistic methods using an internally derived choice of weights give the same estimates as a (multivariable) 2SLS method; the estimates differ for other choices of weights. We investigate the bias and coverage properties of the allele score and summary statistic methods in simulation studies for different choices of weights, in particular with weak instruments. In Section 4, we derive extensions to the previously described methods that can be used when the IVs are correlated and similarly investigate their statistical properties. In Section 5, the methods are illustrated using summarized data on the causal effect of low‐density lipoprotein cholesterol (LDL‐c) on coronary heart disease (CHD) risk, comparing causal estimates obtained using a single genetic variant with those obtained using multiple genetic variants from the same gene region. Finally, we discuss the relevance of these methodological developments to applied practice (Section 6).

For reference, a summary of methods for IV estimation discussed in this paper is given in Table 1. Sample code for implementing the methods is given in Appendix A.1. We clarify that the individual‐level data methods require individual participant data on the genetic variants used as IVs, risk factor and outcome. The summarized data methods only require data on the associations of the IVs with the risk factor and with the outcome. If limited individual‐level data are available (for example, on the IV–risk factor relationship but not the IV–outcome relationship), then summarized associations can be obtained from the individual‐level data, and the analysis can proceed using summarized data only.

**Table 1:** Summary of instrumental variable (IV) estimation methods discussed in this paper.

Method	Equation(s)	Comments
*Individual‐level data methods*		
Two‐stage least squares		Commonly used method in IV analysis (Section 1).
Allele score		Combine IVs into a single score, and use the score as a
		single IV in a two‐stage least squares (or equivalently,
		ratio) method (Section 3.1).
*Summarized data methods (uncorrelated IVs)*		
Allele score	(2) and (3)	The allele score estimate obtained using individual‐level
		data can be approximated using summarized data
		(Section 3.2).
Summary statistic (inverse‐variance weighted)	(4) and (5)	The summary statistic estimate combines the estimates
		from each IV in an inverse‐variance weighted formula
		(Section 3.3). This estimate can also be motivated by
		weighted linear regression through the origin using the
		precisions of the IV associations with the outcome as
		weights.
Likelihood‐based method	(6)	The likelihood‐based method fits a model for the
		summarized data using either maximum likelihood or
		Bayesian methods for inference (Section 3.4).
*Summarized data methods (correlated IVs)*		
Allele score	(2) and (8)	The allele score estimate with summarized data is not
		affected by correlation between the IVs; although the
		estimate's precision is altered (Section 4.1).
Summary statistic (inverse‐variance weighed)	(4) and (9)	With correlated variants, the summary statistic
		formula can be used to test for a causal effect (although
		the standard error of the expression must be modified,
		Section 4.2), but it does not provide an estimate of the
		causal effect.
Weighted generalized linear regression	(10) and (11)	With correlated variants, a weighting matrix can be
		obtained using the standard errors of the IV associations
		with the outcome and the correlations between the
		variants. The coefficient from weighted generalized
		linear regression using this weighting matrix provides
		an estimate of the causal effect (Section 4.2).
Likelihood‐based method	(A1)	Correlation between summarized estimates can be
		incorporated into the likelihood model for the
		summarized data. (Appendix A.3).

## 2 Modelling assumptions

In this paper, the situation of a continuous risk factor and a continuous outcome will be assumed, although the binary outcome case can be handled in a similar way. We assume that the causal effect of the risk factor on the outcome is linear and homogeneous in the population without effect modification. We also assume that the associations of the IVs with the risk factor are linear and homogeneous in the population without effect modification. As shown previously, these assumptions lead to the identification of the causal effect 6. These strong assumptions are not necessary for the estimation of a causal effect; alternative assumptions, such as monotonicity of the IV–risk factor association or no additive effect modification of the causal effect across levels of the instrument at different values of the risk factor, are able to identify a causal parameter 19. However, there is no guarantee that these weaker assumptions will ensure that the same causal effect is estimated by all IVs, particularly for the monotonicity assumption, which identifies a local average treatment effect 20. Hence, weaker assumptions may be tenable in some cases, but the homogeneity assumption is made in this paper.

If the IV–risk factor and IV–outcome associations are estimated in different datasets (known as two‐sample Mendelian randomization 21), we assume that these datasets are sampled from the same underlying population, such that the true association and causal parameters are equal in both datasets. We assume that association estimates used in Mendelian randomization analyses are not conditional on any covariates. If the outcome is continuous, then adjustment for covariates should not affect estimates asymptotically, provided that adjustment is performed uniformly across genetic variants, the covariates are not on the causal pathway from the IV to the outcome, and the IVs remain valid after conditioning on the covariates (so, for example, each IV is independent of confounders conditional on the covariates). If the outcome is binary and association estimates are obtained via logistic regression, then adjustment for covariates will affect estimates asymptotically as coefficients from logistic regression are non‐collapsible 22. However, this should not affect the validity of causal findings, provided that the IVs are valid both marginally and conditionally on the covariates. In particular, adjustment for baseline covariates (such as age and sex) should not be an issue. A full discussion on adjustment of covariates in IV analysis is beyond the scope of this manuscript; further information is available elsewhere 23.

Although these assumptions are restrictive, we note that even if these parametric assumptions are not satisfied, a Mendelian randomization investigation can still be interpreted as a test of the causal null hypothesis, even if the magnitude of the causal effect estimate does not have an interpretation 24, 25. Hence, while these assumptions are necessary to ensure the same causal effect parameter is identified by all IVs, and so that the methods provide consistent estimates of a causal parameter (even in a two‐sample setting), causal inferences from the methods (that is, rejection or otherwise of the null hypothesis of no causal effect) are valid under much weaker assumptions. A causal estimate is nevertheless necessary to combine evidence on the causal effect across multiple IVs. However, the causal estimate could be regarded as a test statistic rather than an estimate. Causal estimates from Mendelian randomization in practice should not be regarded too literally, for example, because different mechanisms for intervention on the same risk factor are likely to lead to different magnitudes of causal effect 5.

Practical issues with respect to the choice of datasets for two‐sample Mendelian randomization are discussed elsewhere 18. In brief, participants in the two datasets should be as similar as possible, for example, with regard to ethnic origin, as otherwise it is more likely that the IV assumptions are invalid in one or other of the datasets. The reason for the particular emphasis on ethnic origin is that genetic variants used in Mendelian randomization are often not the ‘causal’ variant but rather are correlated with the true functional variant through linkage disequilibrium. As linkage disequilibrium patterns often differ between ethnic groups, it would seem prudent to ensure that associations were measured in ethnically homogeneous populations as far as possible. Additionally, if the minor allele frequencies of variants differ between ethnic groups (or other distinct populations or subpopulations), population stratification may bias results 26. In publicly available data from genome‐wide association studies, it is common to adjust for genome‐wide principal components to reduce the influence of population stratification 27. This adjustment generally has a large cumulative effect on association estimates across the genome but a small effect on the association estimates of individual variants. It therefore should not affect association estimates substantially. Hence, although the inclusion of participants of different ethnicities does not necessarily violate the IV assumptions, in such a case, special care should be taken to ensure that the IV assumptions are satisfied in participants of all ethnicities and that the magnitudes of associations and the frequencies of alleles are similar in all subpopulations.

## 3 Uncorrelated instrumental variables

Initially, we consider the scenario where the IVs are uncorrelated.

### 3.1 Individual‐level data allele score method

Most genetic variants used as IVs in Mendelian randomization are biallelic single nucleotide polymorphisms (SNPs) that can be represented as random variables taking the values 0, 1 or 2, denoting the number of risk factor‐increasing alleles in the genotype of an individual. An unweighted allele score is constructed as the total number of risk factor‐increasing alleles for an individual across multiple genetic variants. If an individual *i* has *g*_*ik*_ copies of the risk factor‐increasing allele for each genetic variant *k* = 1,…,*K*, then their unweighted score is $z_{i}=\overset{K}{\underset{k=1}{\sum }}g_{\mathrm{ik}}$. This score takes integer values between 0 and 2*K*. A weighted score can also be considered, in which each variant contributes a weight reflecting the effect of the corresponding genetic variant on the risk factor. If the weight for variant *k* is *w*_*k*_, then individual *i* has a weighted score $z_{i}=\overset{K}{\underset{k=1}{\sum }}w_{k}g_{\mathrm{ik}}$. Provided that the genetic variants that comprise the score are valid IVs, either score can then be used in an IV analysis. Weights are typically taken as estimates of the associations of each IV in turn with the risk factor, obtained from univariate linear regression analyses. These associations may be estimated in the data under analysis, or in an independent dataset.

If the weights in an allele score are derived from the data under analysis, then they will be the same asymptotically as the coefficients from a multivariable regression of the risk factor on the IVs (under the assumption that the IVs are uncorrelated). Values of the weighted score for each individual would therefore equal the fitted values of the risk factor from that regression (up to an additive constant), meaning that the allele score and (multivariable) 2SLS estimates would coincide 12. In this case, the allele score estimate would suffer from the same weak instrument bias as the 2SLS estimate, and there is no benefit in using the allele score method. Better approaches are to estimate the weights using a cross‐validation or jackknife approach 28, to pre‐specify the weights using an external data source, or else (particularly if the variants have approximately equal effects on the risk factor) to use an unweighted score 12.

Under weak instrument asymptotics (the strength of instruments as measured by the concentration parameter – the expected value of the *F* statistic from regression of the risk factor on the IVs – remains fixed as the sample size increases), confidence intervals (CIs) from the 2SLS method using standard asymptotic approximations are overly narrow and coverage rates are below nominal levels 29. Under conventional asymptotics (the strength of instruments increases as the sample size increases), the 2SLS estimator is the most efficient combination of the ratio estimates based on the individual IVs 8, page 553], and coverage rates should tend towards nominal levels. If the weights in an allele score method tend towards the true associations of the IVs with the risk factor, then the allele score estimate will be as efficient asymptotically as the 2SLS estimate. If the weights do not tend towards the true associations, and in particular for an unweighted score, the allele score estimate will be asymptotically inefficient. However, if the true weights of all the IVs are similar, then an unweighted analysis may be more efficient than a weighted analysis in finite samples, as previously demonstrated in a simulation study 12.

### 3.2 Summarized data allele score method

We assume the context of a one‐sample IV analysis in a single dataset with data on the risk factor (*X*), outcome (*Y*) and IVs (*G*_1_,…*G*_*K*_) in all participants. We assume that the estimate of association for IV *k* = 1,…,*K* with the risk factor is ${\hat{\beta }}_{\mathrm{Xk}}$ with standard error *σ*_*Xk*_, and the estimate of association with the outcome is ${\hat{\beta }}_{\mathrm{Yk}}$ with standard error *σ*_*Yk*_. These estimates are typically obtained from linear regression (or logistic regression for associations with a binary outcome). Although the standard errors are estimated, we assume that they are known without error. This may lead to slightly overprecise estimates, but coverage levels have been shown to be close to nominal levels in realistic simulations 17. With the weighted allele score ( $Z=\underset{k}{\sum }w_{k}G_{k}$) used as a single IV, and writing cov for the sample covariance and var for the sample variance, the IV estimate is

(1) $$\begin{array}{cc} \frac{\text{cov}(Y,Z)}{\text{cov}(X,Z)} & =\frac{\text{cov}(Y,\underset{k}{\sum }w_{k}G_{k})}{\text{cov}(X,\underset{k}{\sum }w_{k}G_{k})}\\ =\frac{\underset{k}{\sum }w_{k}\text{cov}(Y,G_{k})}{\underset{k}{\sum }w_{k}\text{cov}(X,G_{k})}\\ =\frac{\underset{k}{\sum }w_{k}{\hat{\beta }}_{\mathrm{Yk}}\mathrm{var}(G_{k})}{\underset{k}{\sum }w_{k}{\hat{\beta }}_{\mathrm{Xk}}\mathrm{var}(G_{k})} \end{array}$$

as the association estimates are calculated as ${\hat{\beta }}_{\mathrm{Yk}}=\text{cov}(Y,G_{k})/\mathrm{var}(G_{k})$ for each *k* = 1,…,*K* (similarly for each ${\hat{\beta }}_{\mathrm{Xk}}$). The weights *w*_*k*_ are assumed to be pre‐specified and are typically taken as the association estimates of each IV with the risk factor in an independent dataset. If the IVs explain a small proportion of variance in the outcome, then var(*G*_*k*_) is approximately proportional to $\sigma_{\mathrm{Yk}}^{-2}$, and so the allele score estimate based on summarized data ( ${\hat{\beta }}_{\mathrm{SSw}}$) is

(2) $${\hat{\beta }}_{\mathrm{SSw}}=\frac{\underset{k}{\sum }w_{k}{\hat{\beta }}_{\mathrm{Yk}}\sigma_{\mathrm{Yk}}^{-2}}{\underset{k}{\sum }w_{k}{\hat{\beta }}_{\mathrm{Xk}}\sigma_{\mathrm{Yk}}^{-2}}$$

We note that at no point in this calculation have we made use of the fact that the genetic variants are uncorrelated. With equal weights, this is equivalent to performing separate inverse‐variance weighted meta‐analyses of the genetic associations with the outcome and of the genetic associations with the risk factor (as the $\sigma_{\mathrm{Xk}}^{-2}$ parameters are approximately proportional to $\sigma_{\mathrm{Yk}}^{-2}$) and then taking the ratio of the pooled estimates. Even in this unweighted case, the directions of the IV associations with the risk factor are required to be specified, even if the magnitudes of the associations are unknown. There have been reports of genetic variants having different directions of association with a risk factor in different datasets 30; however, the majority of these instances were in populations of different ethnic origins, emphasizing the need to use ethnically homogeneous populations in Mendelian randomization and in two‐sample analysis in particular.

The asymptotic standard error of the allele score estimate with uncorrelated variants (equation (2)) can be approximated from summarized data using a delta method 31:

(3) $$\text{se}({\hat{\beta }}_{\mathrm{SSw}})=\sqrt{\frac{\sum \overset{2}{\underset{k}{w}}\overset{-2}{\underset{\mathrm{Yk}}{\sigma }}}{\left(\sum w_{k}{\hat{\beta }}_{\mathrm{Xk}}\sigma_{\mathrm{Yk}}^{-2}\right){}^{2}}+\frac{\left(\sum w_{k}{\hat{\beta }}_{\mathrm{Yk}}\sigma_{\mathrm{Yk}}^{-2}\right){}^{2}\left(\sum \overset{2}{\underset{k}{w}}\overset{-2}{\underset{\mathrm{Yk}}{\sigma }}\right)}{\left(\sum w_{k}{\hat{\beta }}_{\mathrm{Xk}}\sigma_{\mathrm{Yk}}^{-2}\right){}^{4}}-2\theta_{S}\frac{\sum w_{k}{\hat{\beta }}_{\mathrm{Yk}}\sigma_{\mathrm{Yk}}^{-2}}{\left(\sum w_{k}{\hat{\beta }}_{\mathrm{Xk}}\sigma_{\mathrm{Yk}}^{-2}\right){}^{3}}}$$

where *θ*_*S*_ is the correlation between the numerator and denominator in equation (2). This correlation can be estimated by bootstrapping with individual‐level data, or else specified as the observed correlation between the risk factor and the outcome (a sensitivity analysis for the value is advised). In two‐sample Mendelian randomization, this correlation is zero. If the genetic associations with the risk factor are precisely estimated, then the first term will dominate this expression.

### 3.3 Summary statistic (inverse‐variance weighted) method

The summary statistic estimate is calculated using summarized data on the associations of each IV with the risk factor and with the outcome. If the estimates are taken from the same individuals, this is a one‐sample IV analysis; if the estimates are from non‐overlapping groups, this is a two‐sample analysis 21. The ratio method estimate of the causal effect of the risk factor on the outcome using IV *k* is $\frac{{\hat{\beta }}_{\mathrm{Yk}}}{{\hat{\beta }}_{\mathrm{Xk}}}$. The asymptotic standard error of this estimate, derived from the first term of the delta method expansion for the ratio of two random variables 31, is $\frac{\sigma_{\mathrm{Yk}}}{{\hat{\beta }}_{\mathrm{Xk}}}$.

Using the formula for combining estimates in a fixed‐effect inverse‐variance weighted meta‐analysis 32, the summary statistic estimate ${\hat{\beta }}_{\mathrm{SSt}}$ can be calculated as

(4) $${\hat{\beta }}_{\mathrm{SSt}}=\frac{\underset{k}{\sum }{\hat{\beta }}_{\mathrm{Xk}}{\hat{\beta }}_{\mathrm{Yk}}\sigma_{\mathrm{Yk}}^{-2}}{\underset{k}{\sum }{\hat{\beta }}_{\mathrm{Xk}}^{2}\sigma_{\mathrm{Yk}}^{-2}}$$

The approximate asymptotic standard error of the summary statistic estimate is

(5) $$\text{se}({\hat{\beta }}_{\mathrm{SSt}})=\sqrt{\frac{1}{\underset{k}{\sum }{\hat{\beta }}_{\mathrm{Xk}}^{2}\sigma_{\mathrm{Yk}}^{-2}}}$$

This method was previously referred to as an ‘inverse‐variance weighted’ method 17; this refers to the weights in the meta‐analysis formula rather than the weights in the allele score. This estimate can also be motivated as the coefficient from a weighted linear regression of the ${\hat{\beta }}_{\mathrm{Yk}}$ on the ${\hat{\beta }}_{\mathrm{Xk}}$ without an intercept term, using the $\sigma_{\mathrm{Yk}}^{-2}$ as weights. The standard error from an inverse‐variance weighted linear regression in conventional statistical software is often incorrect and has to be modified by forcing the residual standard error to be unity; this can be achieved by dividing the reported standard error by the residual standard error in the regression analysis 33.

If the weights *w*_*k*_ in equation (2) are set to ${\hat{\beta }}_{\mathrm{Xk}}$, then the summary statistic estimate ${\hat{\beta }}_{\mathrm{SSt}}$ equals the allele score estimate using summarized data ${\hat{\beta }}_{\mathrm{SSw}}$, as previously noted 16. In this case, the standard error in equation (5) equals the first term in equation (3). For other choices of weights, the estimates and standard errors will differ.

### 3.4 Likelihood‐based method

A likelihood‐based method has also been proposed, in which the IV associations with the risk factor and with the outcome for each IV are modelled directly by a bivariate normal distribution, with correlation *θ*_*L*_ assumed to be the same for each IV:

(6) $$\left(\begin{array}{l} {\hat{\beta }}_{\mathrm{Xk}}\\ {\hat{\beta }}_{\mathrm{Yk}} \end{array}\right)∼N_{2}\left(\left(\begin{array}{l} \xi_{k}\\ \beta_{L}\xi_{k} \end{array}\right),\left(\begin{array}{ll} \sigma_{\mathrm{Xk}}^{2} & \theta_{L}\;\sigma_{\mathrm{Xk}}\;\sigma_{\mathrm{Yk}}\\ \theta_{L}\;\sigma_{\mathrm{Xk}}\;\sigma_{\mathrm{Yk}} & \sigma_{\mathrm{Yk}}^{2} \end{array}\right)\right)$$

where *β*_*L*_ is the causal parameter. The IV–risk factor association estimates ${\hat{\beta }}_{\mathrm{Xk}}$ are the implicit ‘weights’ in this method. They could be obtained from the dataset under analysis or from an independent dataset (a two‐sample analysis). The standard errors of the association estimates *σ*_*Xk*_ and *σ*_*Yk*_ are used to specify the variance–covariance matrix for the normal distribution and as before are assumed to be known. Model parameters (*β*_*L*_ and *ξ*_*k*_,*k* = 1,…,*K*) can be estimated either by numerical maximization of the log‐likelihood function or in a Bayesian framework 34. Standard errors for maximum‐likelihood estimates can be obtained using the inverse‐Hessian matrix.

The correlation *θ*_*L*_ is due to the IV associations with the risk factor and with the outcome being estimated in the same data. There is likely to be little information on this parameter in the data 35, and so it may be best specified in the analysis as the observational correlation between the risk factor and outcome; a sensitivity analysis can be performed to assess the effect of varying the parameter value on the causal estimate. In a two‐sample IV analysis, the correlation *θ*_*L*_ will be zero.

### 3.5 Simulation study

We investigate the properties of estimates from these methods in a simulation study with two specific goals. The first is to demonstrate that estimates from the allele score method are similar whether they are calculated using individual‐level or summarized data. The second is to compare the behaviour of the summarized data methods (allele score, summary statistic and likelihood‐based) in a two‐sample setting with external weights. We have previously shown that the summary statistic and likelihood‐based methods give similar estimates and standard errors in a one‐sample setting 17. If individual‐level data were available in a one‐sample setting, several other IV methods that are robust to weak instruments could be used, such as limited information maximum likelihood and the continuous updating estimator 36. However, we are unaware of extensions of these methods to a two‐sample setting.

Data on 5000 individuals were generated from the following model in which, for subject *i*, *x*_*i*_ is the risk factor of interest, *u*_*i*_ a confounder, *y*_*i*_ the outcome, and *g*_*ik*_=0,1,2 is the *k*th IV (*k* = 1,…,*K*), representing the number of risk factor‐increasing alleles of a genetic variant:

(7) $$\begin{array}{cc} g_{\mathrm{ik}} & ∼\text{Binomial}(2,\pi_{k})\;\text{independently for}\;k=1,\ldots ,K\\ x_{i} & =\overset{K}{\underset{k=1}{\sum }}\alpha_{k}g_{\mathrm{ik}}+u_{i}+\varepsilon_{\mathrm{Xi}}\\ y_{i} & =\beta_{X}x_{i}+\beta_{U}u_{i}+\varepsilon_{\mathrm{Yi}} \end{array}$$

$$\left.\begin{array}{ll} u_{i} & ∼N(0,1),\varepsilon_{\mathrm{Xi}}∼N(0,1),\varepsilon_{\mathrm{Yi}}∼N(0,1)\\ \pi_{k} & ∼\text{Uniform}(0.01,0.5)\\ \alpha_{k} & ∼\text{Uniform}(0.5\alpha ,1.5\alpha ) \end{array}\right\}\;\text{independently}$$

The causal effect of the risk factor on the outcome is taken as *β*_*X*_=0.2 throughout. The risk factor‐increasing allele frequency *π*_*k*_ and strength of association of the *k*th IV with the risk factor *α*_*k*_ are allowed to vary between the IVs. We set *α* = 0.05,0.1,0.2, and consider scenarios for *K* = 15 IVs with positive (*β*_*U*_=+1) and negative (*β*_*U*_=−1) confounding. As genetic variants are defined arbitrarily with respect to either the risk factor‐increasing or risk factor‐decreasing allele, the restriction to consider only positive values of *α*_*k*_ does not result in any loss of generality. The mean proportion of variance in the risk factor explained by the IVs varies from 1.0% to 10.2%, corresponding to mean *F* statistics from 3.3 to 37.9. 10000 simulations were undertaken for each set of parameter values.

In addition to crude weights (weights estimated naively from the data under analysis using univariate regression of the risk factor on each IV in turn, $w_{k}={\hat{\beta }}_{\mathrm{Xk}}$) and equal weights (*w*_*k*_=1), we also consider external weights, corresponding to a two‐sample IV analysis. The external weight for the *k*th IV is generated by sampling from a normal distribution with mean *α*_*k*_ and variance $\frac{1}{N\pi_{k}(1-\pi_{k})}$. This is equivalent to estimating genetic associations with the risk factor in a separate dataset of size *N* generated under the same model (7). Although in practice, the sample size used for obtaining external weights is often larger than that for the main analysis, the weights will be obtained in a slightly different study population and so may not be entirely appropriate for the data under analysis. Less appropriate weights can be modelled by simulating additional random error in the weights (or equivalently by using a smaller sample size), although in practice there may be systematic as well as random differences between external weights from the first dataset and the true IV–risk factor associations in the second dataset.

All simulations were performed in R 37; sample code is given in Appendix A.1. The allele score method was performed using the tsls command in the *sem* package 38; the summary statistic method was calculated ‘by hand’, and the likelihood‐based method was implemented in a maximum likelihood framework, using the optim command for numerical optimization.

### 3.6 Results

**(a) Comparison of allele score methods**

We calculated the allele score IV estimates for a crudely weighted, equally weighted and externally weighted (*N* = 5000) score using individual‐level data and using summarized data on the genetic associations with the risk factor and outcome as in equation (2). Median estimates and median standard errors across simulations are given in Table 2. The median estimate from an observational analysis (ordinary least squares regression) is also given to judge the direction of confounding. The Monte Carlo standard error, representing the variability in simulation results due to the number of simulated datasets analysed, is 0.002 for the median estimates when *α* = 0.05 and is reduced for larger values of *α*. We additionally provide mean estimates and mean standard errors for this simulation in Table A1; median estimates are preferred as they are not influenced by extreme values and so are more representative of the estimate that may be expected in a typical example. Additionally, the first moment of the ratio IV estimator is formally undefined, so there is a small but finite probability that an allele score estimate takes an arbitrarily large value 39.

**Table 2:** Comparison of allele score methods for uncorrelated IVs.

	*α*	*R* ^2^	*F*	OLS	Crudely weighted	Equally weighted	Externally weighted
					Using individual‐level data		
*β*_*U*_=+1	0.05	0.010	3.3	0.697	0.346 (0.136)	0.198 (0.178)	0.199 (0.205)
	0.10	0.030	10.2	0.687	0.246 (0.080)	0.199 (0.089)	0.198 (0.090)
	0.20	0.102	37.9	0.650	0.212 (0.042)	0.199 (0.044)	0.199 (0.043)
*β*_*U*_=−1	0.05	0.010	3.3	−0.297	0.052 (0.135)	0.201 (0.178)	0.200 (0.205)
	0.10	0.030	10.2	−0.287	0.151 (0.080)	0.198 (0.089)	0.198 (0.090)
	0.20	0.102	37.9	−0.250	0.186 (0.042)	0.199 (0.044)	0.199 (0.043)
					Using summarized data		
*β*_*U*_=+1	0.05	0.010	3.3	0.697	0.346 (0.171)	0.198 (0.204)	0.199 (0.235)
	0.10	0.030	10.2	0.687	0.246 (0.093)	0.199 (0.101)	0.198 (0.102)
	0.20	0.102	37.9	0.650	0.212 (0.048)	0.199 (0.050)	0.199 (0.049)
*β*_*U*_=−1	0.05	0.010	3.3	−0.297	0.052 (0.133)	0.201 (0.168)	0.200 (0.194)
	0.10	0.030	10.2	−0.287	0.151 (0.076)	0.198 (0.083)	0.198 (0.084)
	0.20	0.102	37.9	−0.250	0.186 (0.039)	0.199 (0.042)	0.199 (0.041)

Median estimates over 10 000 simulations of *β*_*X*_=0.2 (median standard errors) from simulation study with 15 uncorrelated instrumental variables (IVs) varying direction of confounding (*β*_*U*_) as shown by median observational estimate (OLS) and average strength of IV (*α*; strength is also expressed by the mean values of the *R* ^2^ and *F* statistics), using allele score methods with crude weights (derived from the data under analysis), equal weights (unweighted analysis) and external weights (equivalent to estimates derived from an independent sample of equal size to the data under analysis), calculated from individual‐level and summarized data.

Allele score estimates calculated using individual‐level data and summarized data were equal to at least the first three decimal places for almost all simulated datasets. This suggests that the approximations used in calculating the allele score estimate using summarized data (most notably, that $\mathrm{var}(G_{k})\propto \sigma_{\mathrm{Yk}}^{-2}$) are reasonable. Estimates with crude weights showed the same pattern of weak instrument bias as those from a 2SLS method; estimates with equal and external weights were unbiased. Standard errors calculated from summarized data, obtained from equation (3), with positive confounding (*β*_*U*_=+1) were sometimes narrower but on average wider than those calculated from individual‐level data. With negative confounding (*β*_*U*_=−1), median standard errors based on summarized data were considerably smaller. The same phenomenon with the average size of standard errors depending on the direction of confounding has been observed previously 40; see Figure 3 of that reference for a potential explanation. Standard errors using summarized data may be improved by including further terms from the delta expansion into equation (3). These simulations are repeated in Table A3 for *K* = 5 and *K* = 25 IVs to investigate the behaviour of these estimates across different numbers of IVs; similar results were observed.

**(b) Comparison of summarized data methods**

Table 3 provides the median estimate, standard deviation of estimates, median standard error, coverage of the 95% CI and empirical power to detect a causal effect based on the nominal 95% CI for the allele score (calculated using summarized data, equation (2)), summary statistic (equation (4)) and likelihood‐based (equation (6)) methods, using external weights based on an independent sample of *N*= 5000 (imprecise weights), 50 000 (precise weights) and using the true ‘oracle’ weights. The oracle weights are the *α*_*k*_ parameters, corresponding to a notional sample size of infinity. The Monte Carlo error for the coverage rate is 0.3% in all scenarios and for the empirical power is 0.5% or lower. Estimates are not provided for the likelihood‐based method with oracle weights as the uncertainty in the weights (*σ*_*Xk*_) cannot be expressed; otherwise, we took $\sigma_{\mathrm{Xk}}=\sqrt{\frac{1}{N\pi_{k}(1-\pi_{k})}}$. The likelihood‐based method failed to report a standard error using imprecise weights with *α* = 0.05 for nine of the 10000 simulated datasets when *β*_*U*_=1 and for five datasets when *β*_*U*_=−1; these results are omitted from Table 3. This simulation study was conducted separately from that for Table 2, and so results differ slightly because of random variation. Mean estimates and mean standard errors are provided in Table A2.

**Table 3:** Comparison of summarized data methods for uncorrelated IVs.

		Imprecise external weights (from 5000 individuals)					Precise external weights (from 50000 individuals)					Oracle weights				
	*α*	Median	SD	SE	Coverage	Power	Median	SD	SE	Coverage	Power	Median	SD	SE	Coverage	Power
		Allele score method using summarized data														
*β*_*U*_=+1	0.05	0.200	0.239	0.219	97.3	14.8	0.198	0.192	0.201	97.0	17.6	0.198	0.186	0.197	97.1	18.0
	0.10	0.200	0.093	0.102	97.1	52.1	0.201	0.089	0.098	97.0	55.6	0.201	0.088	0.098	97.0	56.0
	0.20	0.199	0.044	0.049	97.2	97.8	0.199	0.043	0.049	97.2	98.0	0.199	0.043	0.049	97.1	98.1
*β*_*U*_=−1	0.05	0.201	0.232	0.186	93.1	20.6	0.201	0.196	0.165	92.5	24.9	0.201	0.191	0.161	92.5	25.7
	0.10	0.200	0.092	0.084	92.7	68.1	0.200	0.088	0.081	93.3	71.2	0.199	0.087	0.080	93.4	71.7
	0.20	0.200	0.044	0.041	93.0	99.7	0.200	0.043	0.040	92.8	99.8	0.200	0.043	0.040	92.8	99.8
		Summary statistic method^a^														
*β*_*U*_=+1	0.05	0.148	0.168	0.162	93.1	14.8	0.191	0.191	0.187	94.8	17.6	0.199	0.193	0.190	95.0	18.0
	0.10	0.183	0.094	0.091	94.2	52.1	0.199	0.096	0.095	94.8	55.6	0.201	0.096	0.095	95.0	56.0
	0.20	0.194	0.048	0.047	94.9	97.8	0.198	0.048	0.047	95.0	98.0	0.199	0.048	0.047	94.9	98.1
*β*_*U*_=−1	0.05	0.147	0.139	0.133	93.0	20.6	0.194	0.158	0.154	94.7	24.9	0.201	0.161	0.157	94.8	25.7
	0.10	0.181	0.077	0.075	94.0	68.1	0.198	0.078	0.078	95.3	71.2	0.200	0.079	0.078	95.3	71.7
	0.20	0.196	0.040	0.039	94.3	99.7	0.200	0.040	0.039	94.9	99.8	0.200	0.040	0.039	95.0	99.8
		Likelihood‐based methoda														
*β*_*U*_=+1	0.05	0.203	0.242	0.195	94.0	18.3	0.194	0.182	0.191	97.3	17.0					
	0.10	0.201	0.104	0.097	94.4	54.3	0.200	0.096	0.095	95.0	55.6					
	0.20	0.197	0.050	0.048	94.5	97.7	0.198	0.052	0.048	93.4	97.8					
*β*_*U*_=−1	0.05	0.204	0.201	0.161	94.3	25.4	0.198	0.153	0.157	97.1	24.4					
	0.10	0.198	0.086	0.080	94.5	70.1	0.199	0.079	0.079	95.2	71.3					
	0.20	0.199	0.042	0.040	94.3	99.7	0.200	0.046	0.039	92.0	99.7					

Median estimates over 10000 simulations of *β*_*X*_=0.2, standard deviation (SD) of estimates, median standard error (SE) of estimates, coverage (%) of nominal 95% confidence interval for the causal parameter and empirical power (%) based on nominal 95% confidence interval to detect a causal effect from simulation study with 15 uncorrelated instrumental variables (IVs) varying the direction of confounding (*β*_*U*_) and average strength of IV (*α*) using three summarized data methods: allele score, summary statistic and likelihood‐based methods, with weights taken from an external source corresponding to an independent sample of size 5000, 50000 and using the true (oracle) weights.

^a^The ‘weights’ for the summary statistic and likelihood‐based methods are used as the ${\hat{\beta }}_{\mathrm{Xk}}$ association estimates in equations (4) and (6). In the allele score method, the weights (*w*_*j*_) and the association estimates ( ${\hat{\beta }}_{\mathrm{Xk}}$) in the data under analysis are both used to provide the causal estimate.

Allele score estimates using external weights were unbiased, with median estimates close to the true value of 0.2. With positive confounding, coverage levels were conservative compared with the nominal 95% level, whereas with negative confounding, coverage levels were often slightly below nominal levels. With imprecise weights, the summary statistic estimates were biased towards the null; this bias disappeared as the weights become more precise. However, coverage rates were close to nominal levels. Further investigations showed that the summary statistic estimates with external weights were unbiased under the null (Table A4). The slight bias towards the null in the summary statistic estimates with external weights is similar to that observed for the 2SLS method in a two‐sample setting 21, 41. Bias towards the null occurs for the same reason as regression dilution bias in a linear regression model with error in the regressor 42. Estimates from the likelihood‐based method were unbiased, with coverage levels occasionally dropping below the nominal 95% level, particularly with more precise weights. This may be due to lack of convergence in the optimization algorithm.

The summary statistic and allele score methods had equal power estimates and rejected or accepted the null together. The likelihood‐based method dominated the other methods in terms of power with imprecise weights; with precise weights, power was very similar between the methods. The power of estimates increased as weights became more precise, although the increase from using weights estimated in a sample size of 50000 to the oracle weights was not substantial.

### 3.7 Practical implications

It is likely that the choice of weights will have a greater impact on the findings of Mendelian randomization investigations than differences between analysis methods. For choosing weights in practice, we echo the advice of Burgess and Thompson 12: first, internally derived ‘crude’ weights should be avoided; secondly, the source for externally derived weights should be primarily chosen to be relevant to the population under analysis (for example, in terms of ethnicity, sex and age); and thirdly, the external source should be the largest available sample so that the weights are precisely estimated. If the weights are imprecise, then estimates from the summary statistic method will be biased towards the null. Although this bias is conservative, and hence is less serious than weak instrument bias, in this context an unweighted analysis or the summarized data allele score method may be preferred.

## 4 Correlated instrumental variables

The use of multiple genetic variants in a Mendelian randomization analysis is often necessary to give clinically relevant results because of low power. In some circumstances, including multiple potentially correlated variants from a single gene region is likely to lead to a more reliable analysis than one using variants from multiple gene regions. Although including additional genetic variants that are perfectly correlated will not increase the precision of a Mendelian randomization analysis, the inclusion of multiple variants in partial linkage disequilibrium can explain a greater proportion of variance in the risk factor. If the variants explain additional variation in the risk factor, this will lead to more powerful Mendelian randomization analyses. We do not make any assumption on the underlying genetic architecture leading to multiple correlated genetic variants that each explain independent variation in the risk factor but note that a genetic variant is not required to be a ‘causal variant’ for use in Mendelian randomization 43. If there is one genetic variant in a particular gene region that is the single causal variant, and this variant is measured in the dataset, then the use of multiple correlated variants will not add power to the analysis but neither will it invalidate the analysis provided that the additional variants do not violate the IV assumptions. However, it may lead to increased weak instrument bias and decreased efficiency.

Extensions to allow for correlated IVs have been discussed in the context of the likelihood‐based method 18 and can be implemented by allowing for a joint multivariate distribution of the ${\hat{\beta }}_{\mathrm{Xk}}$ and ${\hat{\beta }}_{\mathrm{Yk}}$ estimates, with the variance–covariance matrix incorporating the correlation between the IVs (see Appendix A.3 for details). We proceed to consider extensions to allow for correlated variants in the summarized data allele score and summary statistic methods.

### 4.1 Extension to allele score method with summarized data

An allele score composed of genetic variants that are valid IVs will be a valid IV regardless of correlation between variants. However, if the weights for the score are taken from univariate regression analyses, IV estimates may be inefficient. For instance, if two sets of highly correlated variants have the same strength of association with the risk factor for each genetic variant, taking weights from univariate regression analyses will assign weight overall in proportion to the number of variants measured in each set. A better approach would be to take weights from a multivariable regression of the risk factor on all the IVs in the analysis model. However, unless the weights are obtained in an independent dataset, this is likely to exacerbate weak instrument bias as overfitting is a greater problem when the predictors in a regression model are correlated. Moreover, in practice, it is unlikely that genetic association estimates from a multivariable regression model in an independent dataset would be generally available, and so, we do not pursue complex strategies for weighting allele scores further in this manuscript. We restrict our attention to equally weighted and externally weighted scores, where the external weights are modelled as univariate weights from an independent dataset.

As noted previously, correlation between IVs should not affect allele score estimates based on pre‐specified weights (equation (2)), although it will affect their precisions. The standard error of an allele score with correlated IVs can be approximated using summarized data as

(8) $$\text{se}({\hat{\beta }}_{\mathrm{SSw}})=\sqrt{\frac{\underset{k_{1}}{\sum }\underset{k_{2}}{\sum }\rho_{k_{1}k_{2}}w_{k_{1}}w_{k_{2}}\sigma_{Yk_{1}}^{-1}\sigma_{Yk_{2}}^{-1}}{\left(\underset{k}{\sum }w_{k}{\hat{\beta }}_{\mathrm{Xk}}\sigma_{\mathrm{Yk}}^{-2}\right){}^{2}}}$$

where $\rho_{k_{1}k_{2}}$ is the correlation between IVs *k*_1_ and *k*_2_. Only the first term from the delta method expansion corresponding to equation (3) is presented.

Estimates of correlations between genetic variants can be obtained from the published literature if they are not otherwise available, for example, using the SNP Annotation and Proxy Search (SNAP, http://www.broadinstitute.org/mpg/snap/ldsearch.php) 44. We assume that these correlations are known without error. However, it may be problematic to determine the direction of correlation between two variables from published data alone. Additionally, these estimates are often based on small sample sizes and are only available for a limited number of reference populations.

### 4.2 Extension to summary statistic method

Similarly, if the summary statistic method in equation (4) is used to test the presence of a causal effect, the standard error of this expression is approximately

(9) $$\text{se}\left({\hat{\beta }}_{\mathrm{SSt}}\right)=\sqrt{\frac{\underset{k_{1}}{\sum }\underset{k_{2}}{\sum }\rho_{k_{1}k_{2}}{\hat{\beta }}_{Xk_{1}}{\hat{\beta }}_{Xk_{2}}\sigma_{Yk_{1}}^{-1}\sigma_{Yk_{2}}^{-1}}{\left(\underset{k}{\sum }{\hat{\beta }}_{\mathrm{Xk}}^{2}\sigma_{\mathrm{Yk}}^{-2}\right){}^{2}}}.$$

If the weights *w*_*k*_ in equations (2) and (8) are equal to ${\hat{\beta }}_{\mathrm{Xk}}$, then the test statistic using this standard error, $\frac{{\hat{\beta }}_{\mathrm{SSt}}}{\text{se}({\hat{\beta }}_{\mathrm{SSt}})}$, is exactly the same as the test statistic for the allele score method using summarized data evaluated using equation (8), $\frac{{\hat{\beta }}_{\mathrm{SSw}}}{\text{se}({\hat{\beta }}_{\mathrm{SSw}})}$, and the two methods will therefore reject or not reject the null hypothesis together. However, the expression in equation (4) will not be an estimate of the causal effect as it is affected by correlation between the IVs.

Alternatively, in the same way as the summary statistic method with uncorrelated IVs in equation (4) can be viewed as a weighted linear regression of the ${\hat{\beta }}_{\mathrm{Yk}}$ parameters on the ${\hat{\beta }}_{\mathrm{Xk}}$ parameters with no intercept term, we can perform a weighted generalized linear regression of the ${\hat{\beta }}_{\mathrm{Yk}}$ parameters on the ${\hat{\beta }}_{\mathrm{Xk}}$ parameters using the $\sigma_{\mathrm{Yk}}^{-2}$ parameters as inverse‐variance weights and taking into account the correlation between the IVs.

If $\Omega_{k_{1}k_{2}}=\sigma_{Yk_{1}}\sigma_{Yk_{2}}\rho_{k_{1}k_{2}}$, then the estimate from a weighted generalized linear regression is

(10) $${\hat{\beta }}_{\mathrm{SSc}}=\left({\hat{\beta }}_{\mathrm{Xk}}^{T}\Omega^{-1}{\hat{\beta }}_{\mathrm{Xk}}\right){}^{-1}{\hat{\beta }}_{\mathrm{Xk}}^{T}\Omega^{-1}{\hat{\beta }}_{\mathrm{Yk}}$$

The standard error of the estimate is

(11) $$\text{se}\left({\hat{\beta }}_{\mathrm{SSc}}\right)=\sqrt{\left({\hat{\beta }}_{\mathrm{Xk}}^{T}\Omega^{-1}{\hat{\beta }}_{\mathrm{Xk}}\right){}^{-1}}$$

Unlike with uncorrelated variants, where the summary statistic and weighted linear regression estimates coincide, the weighted generalized linear regression estimate ${\hat{\beta }}_{\mathrm{SSc}}$ does not equal the estimate in equation (4). The weighted generalized linear regression method should provide an estimate of the causal parameter.

### 4.3 Simulation study

We perform a further simulation study to investigate the properties of estimates from these methods with correlated IVs. Other than as specified, all parameters and features of the simulation study are the same as those in the simulation with uncorrelated IVs. As previously, the main goals are to consider the similarity of allele score estimates calculated using individual‐level and summarized data and to compare the behaviour of the summarized data methods (allele score, weighted generalized linear regression and likelihood‐based) with external weights. We do not consider the summary statistic method (equations (4) and (9)), as this does not provide an estimate of the causal effect with correlated variants. In addition to performing simulations with a positive causal effect, we also consider the scenario with a null causal effect. This is because we are particularly concerned that data on correlated IVs should not artificially add precision to IV analyses so that nominal coverage properties are maintained under the null.

The data‐generating model is as follows:

(12) $$\begin{array}{cc} \Lambda & ∼\text{Wishart}(K,\Lambda_{0}),\;\Phi =\text{Cor}(\Lambda )\\ \psi_{1i},\psi_{2i} & ∼N_{K}(0,\Phi )\;\text{independently}\\ g_{\mathrm{ik}} & =1_{\psi_{1\mathrm{ik}}>\pi_{k}^{\prime }}+1_{\psi_{2\mathrm{ik}}>\pi_{k}^{\prime }}\\ x_{i} & =\overset{K}{\underset{k=1}{\sum }}\alpha_{k}g_{\mathrm{ik}}+u_{i}+\varepsilon_{\mathrm{Xi}}\\ y_{i} & =\beta_{X}x_{i}+\beta_{U}u_{i}+\varepsilon_{\mathrm{Yi}} \end{array}$$

$$\left.\begin{array}{ll} u_{i} & ∼N(0,1),\varepsilon_{\mathrm{Xi}}∼N(0,1),\varepsilon_{\mathrm{Yi}}∼N(0,1)\\ \pi_{k}^{\prime } & ∼\text{Uniform}(0,2)\\ \alpha_{k} & ∼\text{Uniform}(0.5\alpha ,1.5\alpha ) \end{array}\right\}\;\text{independently}$$

where Λ_0_ is a matrix parameter that determines the distribution of correlations between genetic variants; it is taken to have diagonal elements 1 and off‐diagonal elements 0.5. The variables ***ψ***_1*i*_ and ***ψ***_2*i*_ are independent vectors of length *K* and represent the two haplotypes of an individual; for each haplotype, if the *k*th component of the vector *ψ*_1*ik*_ or *ψ*_2*ik*_ is greater than a reference value $\pi_{k}^{\prime }$, a risk factor increasing allele is recorded for the *k*th genetic variant in individual *i* 45. Correlations between IVs were generated by simulating a matrix Λ from a Wishart distribution and then normalizing by taking the correlation matrix Φ=Cor(Λ), so that *ψ*_1*ik*_ and *ψ*_2*ik*_ have marginal standard normal distributions for all *k* = 1,…,*K*. If $\pi_{k}^{\prime }=0$, the risk factor increasing allele for genetic variant *k* has frequency 0.5, while if $\pi_{k}^{\prime }=1.96$, the frequency is 0.025. The estimated correlations between IVs ( $\rho_{k_{1}k_{2}}$) were mostly (78%) positive, with an average first quartile of 0.06 and third quartile of 0.30 across all pairwise correlations. A further simulation analyses was also performed (not reported) in which the off‐diagonal elements of Λ_0_ were all 0.2; findings were substantially the same as those reported in this paper.

We considered two values of the causal effect *β*_*X*_=0.2 and *β*_*X*_=0. Rather than generating external weights using a random draw from a normal distribution, we generated independent data from the same data‐generating model for 5000 participants and used univariate regression for each IV in these individuals to derive external weights. This procedure should closely mirror an applied two‐sample analysis, particularly one using published summarized data on genetic associations with the risk factor.

### 4.4 Results

**(a) Comparison of allele score methods**

Table 4 shows the median estimates, median standard errors and power of the nominal 95% CI for the allele score method calculated using individual‐level data and using summarized data (equations (2) and (8)) with equal weights. Mean estimates and mean standard errors are provided in Table A5.

**Table 4:** Comparison of allele score methods for correlated instrumental variables (IVs).

	*α*	*R* ^2^	*F*	Allele score using individual‐level data	Allele score using summarized data
Positive causal effect: *β*_*X*_=0.2					
*β*_*U*_=+1	0.05	0.019	6.3	0.201 (0.116) [43.3]	0.201 (0.129) [34.9]
	0.10	0.062	22.2	0.201 (0.058) [87.2]	0.201 (0.065) [84.2]
	0.20	0.201	85.8	0.200 (0.029) [99.9]	0.200 (0.033) [99.8]
*β*_*U*_=−1	0.05	0.019	6.3	0.202 (0.117) [39.4]	0.202 (0.107) [48.0]
	0.10	0.062	22.2	0.199 (0.058) [91.8]	0.199 (0.053) [93.2]
	0.20	0.201	85.8	0.200 (0.029) [100.0]	0.200 (0.027) [100.0]
Null causal effect: *β*_*X*_=0					
*β*_*U*_=+1	0.05	0.019	6.3	0.001 (0.116) [4.4]	0.001 (0.116) [4.8]
	0.10	0.062	22.2	0.000 (0.058) [4.7]	0.000 (0.058) [4.8]
	0.20	0.201	85.8	0.000 (0.029) [5.1]	0.000 (0.029) [5.0]
*β*_*U*_=−1	0.05	0.019	6.3	−0.002 (0.116) [4.3]	−0.002 (0.117) [4.8]
	0.10	0.062	22.2	0.000 (0.058) [5.0]	−0.001 (0.058) [5.0]
	0.20	0.201	85.8	0.000 (0.029) [5.1]	0.000 (0.029) [5.2]

Median estimates over 10000 simulations of *β*_*X*_=0.2 or *β*_*X*_=0 (median standard errors) [power (%) based on nominal 95% confidence interval] from simulation study with 15 correlated IVs varying direction of confounding (*β*_*U*_) and average strength of IV (*α*; strength is also expressed by the mean values of the *R* ^2^ and *F* statistics) using allele score methods calculated from individual‐level and summarized data, with equal weights.

The pattern of results for the allele score methods is very similar with correlated IVs as with uncorrelated IVs. Estimates in each simulated dataset calculated using individual‐level data and summarized data were equal to at least three decimal places and were unbiased both with a positive causal effect and with a null causal effect. Median standard errors with a positive causal effect using summarized data were slightly larger with positive confounding, and slightly smaller with negative confounding, compared with those with individual‐level data. With a null causal effect, median standard errors were almost the same using individual‐level and summarized data and did not vary with the direction of confounding. The power to detect a causal effect with *β*_*X*_=0 was around 5%, meaning that coverage rates (type I error rates) were at correct nominal levels.

**(b) Comparison of summarized data methods**

Table 5 shows the median estimates, median standard errors and power of the nominal 95% CI for the allele score method calculated using individual‐level data (for comparison) and the allele score method using summarized data, the summary statistic method calculated using weighted generalized regression (equations (10) and (11)) and the likelihood‐based method with correlated IVs; all estimates were obtained with external weights (based on an independent sample size of *N* = 5000). The likelihood‐based method failed to report a standard error with *α* = 0.05 for between 1 and 9 of the 10000 simulated datasets for each set of parameter values and weights; these results were omitted from Table 5. Mean estimates and mean standard errors are given in Table A6.

**Table 5:** Comparison of summarized data methods for correlated instrumental variables (IVs).

	*α*	Allele score using individual‐level data	Allele score using summarized data	Weighted generalized linear regression	Likelihood‐based method
Positive causal effect: *β*_*X*_=0.2					
*β*_*U*_=+1	0.05	0.201 (0.120) [41.5]	0.201 (0.134) [33.2]	0.147 (0.109) [27.6]	0.197 (0.131) [33.2]
	0.10	0.201 (0.059) [85.6]	0.201 (0.066) [82.6]	0.184 (0.061) [81.9]	0.198 (0.066) [83.0]
	0.20	0.200 (0.030) [99.8]	0.200 (0.033) [99.8]	0.195 (0.032) [99.8]	0.190 (0.032) [99.8]
*β*_*U*_=−1	0.05	0.202 (0.121) [36.7]	0.202 (0.111) [45.4]	0.147 (0.090) [38.6]	0.201 (0.109) [45.1]
	0.10	0.199 (0.060) [90.2]	0.199 (0.055) [91.6]	0.182 (0.051) [91.3]	0.194 (0.054) [92.0]
	0.20	0.200 (0.030) [100.0]	0.200 (0.027) [100.0]	0.196 (0.026) [100.0]	0.183 (0.026) [100.0]
Null causal effect: *β*_*X*_=0					
*β*_*U*_=+1	0.05	0.002 (0.120) [4.5]	0.002 (0.120) [5.0]	0.002 (0.098) [4.9]	0.003 (0.114) [6.7]
	0.10	0.000 (0.060) [4.6]	0.000 (0.060) [4.8]	0.000 (0.055) [4.9]	0.000 (0.058) [5.1]
	0.20	−0.001 (0.030) [5.0]	−0.001 (0.030) [5.0]	0.000 (0.029) [4.9]	0.000 (0.029) [4.8]
*β*_*U*_=−1	0.05	0.000 (0.120) [4.5]	0.000 (0.120) [5.0]	−0.001 (0.098) [4.6]	−0.001 (0.114) [6.4]
	0.10	0.000 (0.060) [4.6]	0.000 (0.060) [4.8]	0.000 (0.055) [4.8]	0.000 (0.058) [4.9]
	0.20	0.000 (0.030) [5.1]	0.000 (0.030) [5.3]	0.000 (0.028) [5.2]	0.000 (0.029) [5.0]

Median estimates over 10000 simulations of *β*_*X*_=0.2 or *β*_*X*_=0 (median standard errors) [power (%) based on nominal 95% confidence interval] from simulation study with 15 correlated IVs varying direction of confounding (*β*_*U*_) and average strength of IV (*α*) using allele score method calculated from individual‐level data and allele score, weighted generalized linear regression and likelihood‐based methods all calculated from summarized data, with external (*N* = 5000) weights.

Results for the summarized data methods were similar with correlated IVs as with uncorrelated IVs. Estimates with external weights were unbiased under the null with nominal coverage rates preserved. With a positive causal effect, estimates from the weighted generalized linear regression method were slightly biased towards the null, reflecting the uncertainty in the IV associations with the risk factor. For the likelihood‐based method, there was bias towards the null with the strongest IVs and slight undercoverage under the null with the weakest IVs. Examination of the values of the optimized log‐likelihood function revealed lack of convergence for a small number of datasets, particularly with *β*_*X*_=0.2.

The simulations were also repeated with crude weights (Table A7). With crude weights, estimates from the allele score, weighted generalized linear regression and likelihood‐based methods were biased in the direction of the confounded observational estimate with undercoverage under the null. This was more pronounced for the weighted generalized linear regression and likelihood‐based methods, compared with than the allele score methods. Additionally, further simulations were performed for a binary outcome using external weights with the same parameters and a similar data‐generating mechanism (Table A8). Results were broadly similar to those with a continuous outcome. Estimates were generally unbiased with nominal coverage rates maintained under the null; there was a small positive bias in the weighted generalized linear regression and likelihood‐based methods, although this did not lead to substantial over‐rejection of the null. With a positive causal effect, power to reject the null was reduced compared with a continuous outcome, and the median estimates were attenuated towards the null; this is a known phenomenon and relates to the non‐collapsibility of odds ratios 22, 46. This simulation suggests that the methods presented in this paper will lead to appropriate causal inferences with binary outcomes, although the precise interpretation of the causal estimate is further complicated.

### 4.5 Practical implications

Allele score and weighted generalized linear regression estimates using summarized data showed good statistical properties with correlated genetic variants, particularly under the null hypothesis. With a non‐null causal effect and imprecisely estimated weights, the weighted generalized linear regression estimates were slightly biased towards the null. This suggests that Mendelian randomization analyses can be performed using summarized data on correlated genetic variants, provided that data on the correlations between the variants are available. Although the likelihood‐based method has good theoretical properties, caution should be taken with the method in practice to ensure that the optimization routine has converged appropriately. A sensitivity analysis can be undertaken by repeating the analysis in a Bayesian analysis framework; software code for a two‐sample analysis is provided elsewhere 18.

## 5 Example: effect of LDL‐cholesterol on coronary heart disease risk

To illustrate the methods described earlier, we estimate the causal effect of LDL‐c on CHD risk using genetic variants from the *PCSK9* gene region. We consider rs11206510, previously shown to be associated with LDL‐c concentration and CHD risk 47, as the primary SNP in the analysis. We investigate how including potentially correlated genetic variants located adjacent to the primary SNP influences the precision of findings.

**Figure 1:**
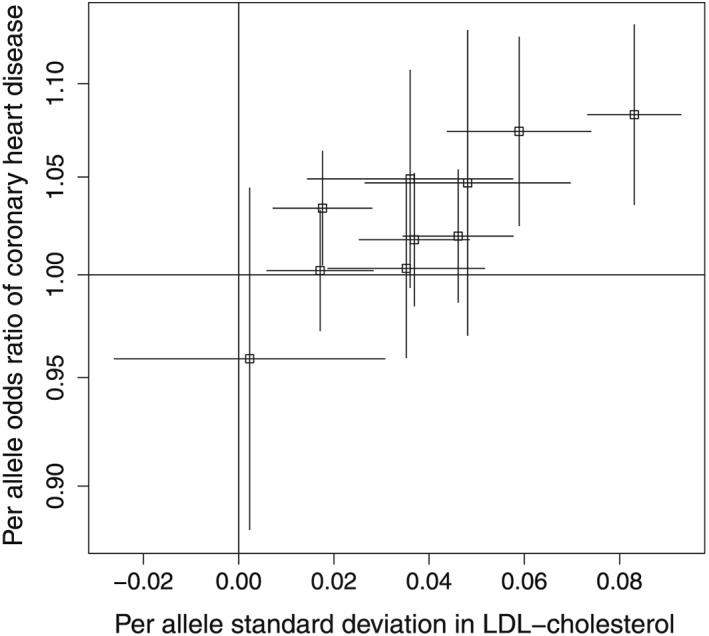
Estimated genetic associations and 95% confidence intervals with low‐density lipoprotein cholesterol (LDL‐c) and with coronary heart disease risk for 10 genetic variants in the *PCSK9* gene region.

Genetic associations with LDL‐c were taken from the GLGC 13 and can be downloaded from http://www.sph.umich.edu/csg/abecasis/public/lipids2013. Genetic associations with CHD risk were taken from the CARDIoGRAM consortium 48 and can be downloaded from http://www.cardiogramplusc4d.org/downloads. Estimates from both GLGC and CARDIoGRAM were obtained using data on individuals of European descent, mostly of working age, and so the two datasets should be similar; in fact, several studies are included in both consortia. After pruning for linkage disequilibrium at *r* ^2^>0.8, all variants in a 10‐kilobase pair region around rs11206510 available in both the GLGC and CARDIoGRAM consortia were included in the analysis; 10 variants in total were included. Associations with LDL‐c and CHD risk are given in Table A9 and displayed graphically in Figure 1. There is an apparent dose–response relationship, with variants associated with greater per allele changes in LDL‐c also having greater odds ratios for CHD. There is no obvious heterogeneity in the causal effects from different individual variants. Correlations between the genetic variants were taken from the SNAP database; it was assumed that all the risk factor increasing alleles were positively correlated. The correlation *θ*_*L*_ in the likelihood‐based method (equation (6)) is taken as zero.

The causal effect estimate, representing the odds ratio of CHD per 1 standard deviation increase in LDL‐c, calculated using the summary statistic method of equations (4) and (5) based on the primary SNP rs11206510 alone, was 2.62 (95% CI: 1.52, 4.49). The corresponding causal estimate based on all the genetic variants, ignoring correlations between the variants, was 2.25 (95% CI: 1.65, 3.07) – this estimate is overly precise and the CI is too narrow. Accounting for the correlations, using the weighted generalized linear regression method of equations (10) and (11), the causal estimate was 2.28 (95% CI: 1.53, 3.38). Using the allele score method for correlated variants of equations (2) and (8) with the published univariate association estimates as weights, the causal estimate was 2.25 (95% CI: 1.41, 3.59); with equal weights, the causal estimate was 2.14 (95% CI: 1.18, 3.86). Using the likelihood‐based method for correlated variants, the causal estimate was 2.31 (95% CI: 1.53, 3.50). These results are additionally presented in Table 6. The *p*‐value from Cochran's Q statistic of heterogeneity in the causal estimates for each genetic variant calculated individually was 0.53, indicating no more heterogeneity between causal effects estimated using the variants individually than would be expected by chance.

**Table 6:** Estimates and 95% confidence intervals (CI) of causal effect of low‐density lipoprotein‐cholesterol on coronary heart disease risk using genetic variants from *PCSK9* gene region from various analysis methods.

Method	Equations	Estimate	95% CI
Estimate based on single genetic variant (rs11206510)	(4) and (5)	2.62	1.52, 4.49
Summary statistic method based on all genetic variants	(4) and (5)	2.25	1.65, 3.07
ignoring correlation			
Weighted generalized linear regression method based	(10) and (11)	2.28	1.53, 3.38
on all genetic variants incorporating correlation			
Allele score method based on all genetic variants	(2) and (8)	2.25	1.41, 3.59
incorporating correlation using estimated weights			
Allele score method based on all genetic variants	(2) and (8)	2.14	1.18, 3.86
incorporating correlation using equal weights			
Likelihood‐based method based on all genetic	See Appendix A.3	2.31	1.53, 3.50
variants incorporating correlation			

Overall, the results from the applied example were similar to those from the simulation analyses. Estimates using all the variants were more precise than those only using the lead variant, with a relative efficiency of 186% based on the summary statistic method results. Under the assumption that the variances of estimates are inversely proportional to the sample size, an equivalent gain in precision in the single SNP analysis could be achieved by increasing the sample size for the genetic associations with the outcome by 86%. Out of methods accounting for the correlation between variants, the estimate from the weighted generalized linear regression method had the narrowest CI, followed by the likelihood‐based method and then the allele score method using external weights. The point estimate from the allele score method using equal weights was similar to those from other methods, but the CI was wider, reflecting the different magnitudes of association of the genetic variants with the risk factor.

By including more genetic variants from in and around this gene region, more precise causal estimates were obtained. As the genetic associations with the risk factor and with the outcome are estimated in samples with little substantial overlap, it is likely that any bias due to weak instruments would be in the direction of the null 21.

## 6 Discussion

Much information useful for performing Mendelian randomization studies is now available in the form of summarized data. In this paper, we have provided formulae for calculating an allele score estimate with arbitrarily chosen weights using summarized data on genetic associations with the risk factor and with the outcome. This enables allele score estimates using equal or external weights to be calculated without requiring individual‐level data. The allele score estimate using crude weights (those calculated from the data under analysis) in a one‐sample setting is approximately equal to a commonly used summary statistic (inverse‐variance weighted) estimate. This summary statistic estimate is equivalent to an estimate from a weighted linear regression analysis. Both the allele score (calculated either using individual‐level or summarized data) and summary statistic estimates with crude weights are approximately equal to an estimate from a 2SLS method. This means that allele score and summary statistic estimates using crude weights suffer from weak instrument bias and are biased in the direction of the observational association. In contrast, allele score and summary statistic estimates using equal or externally derived weights give valid tests of the null hypothesis of no causal effect. Estimates from the summary statistic method using external weights are conservatively biased towards the null when the external weights are imprecise estimates of the true weights.

We have also provided formulae for calculating an allele score estimate using summarized data with correlated IVs, which in Mendelian randomization correspond to genetic variants in linkage disequilibrium. Alternatively, a causal estimate can be calculated from summarized data using weighted generalized linear regression. These methods enable researchers to perform IV analyses with correlated IVs using summarized data and in particular Mendelian randomization analyses with more than one genetic variant in a given gene region. If the multiple variants explain more of the variance in the risk factor than any single variant, then power to detect a causal effect will be improved. Inclusion of multiple genetic variants from a single gene region may provide a better way of improving power in Mendelian randomization investigations than inclusion of genetic variants from multiple gene regions, as variants from a single candidate region may be more likely to satisfy the IV assumptions.

There are several practical considerations to take into consideration when using summarized data. If the IV assumptions are violated even for one genetic variant in a Mendelian randomization analysis, then causal estimates will be biased and type I error rates will be inflated, as previously demonstrated in the context of allele scores 12. It will not be possible to assess the IV assumptions as rigorously or as systematically in summarized data as in individual‐level data. However, many analyses for assessing the validity of the IV assumptions are still possible. If the coefficient for the association of an SNP with the risk factor ${\hat{\beta }}_{\mathrm{Xk}}$ is given in standard deviation units, then the proportion of variance in the risk factor explained by the SNP (the *R* ^2^ statistic) is approximately equal to $2{\hat{\beta }}_{\mathrm{Xk}}^{2}\times \mathrm{MAF}\times (1-\mathrm{MAF})$, where *MAF* is the minor allele frequency. The *F* statistic can then be calculated from the *R* ^2^ statistic as $F=\frac{N-K-1}{K}\frac{R^{2}}{1-R^{2}}$, where *N* is the sample size and *K* is the number of genetic variants. An overidentification test can be performed by considering the ratio estimates from each IV individually and performing a heterogeneity test, such as Cochran's Q test 49. A test for directional pleiotropy based on summarized data has also been proposed 50; this considers a weighted linear regression of the ${\hat{\beta }}_{\mathrm{Yk}}$ on the ${\hat{\beta }}_{\mathrm{Xk}}$ that is similar to the one discussed in this paper but with an intercept term. The intercept term represents the average association of an IV with the outcome in the absence of association with the risk factor. Under the IV assumptions that the association of each IV with the outcome is mediated via the risk factor, this intercept term should be zero. If the estimated intercept term is different from zero, there is evidence of direct effects of IVs on the outcome not via the risk factor that do not average out; this is known as directional pleiotropy.

As a recommendation for using summarized data, we suggest either the allele score or weighted (generalized) linear regression methods. If the likelihood‐based method is used, care should be taken to check whether the optimization algorithm has converged appropriately. The allele score method is desirable as estimates are unbiased with either equal or externally derived weights, and the summary statistic method has good intuitive justification from weighted linear regression, although there is some bias towards the null with imprecise externally derived weights. The formulae provided in this paper mean that evaluating a causal estimate based on multiple IVs that does not suffer from weak instrument bias is relatively simple; ensuring that the choice of IVs and the interpretation of the causal analysis are appropriate is the difficult step.

## Appendix 1

### 1.1 A.1 Sample code

Here, we provide sample code written in R 37 to implement the methods used in this paper.

The allele score method using individual‐level data was performed using the tsls command in the *sem* package:

The summary statistic (inverse‐variance weighted) method for uncorrelated IVs (equation (4)) can be calculated using the IV associations with the risk factor (bx, standard error bxse) and the IV associations with the outcome (by, standard error byse). The IV associations with the risk factor may be taken from the data under analysis (one‐sample analysis), or from an external data source (two‐sample analysis):

The same summary statistic (inverse‐variance weighted) estimate can be obtained using weighted linear regression. We recall that the standard error of the causal estimate has to be modified by forcing the residual standard error in the regression model to be unity; this can be achieved by dividing the reported standard error by the residual standard error in the regression analysis 33:

A heterogeneity test based on Cochran's Q statistic can be performed using the summarized data. We use the metagen command, from the package *meta*. This assesses the similarity of the causal effects from each IV (by/bx), given estimates of the uncertainty of the IV estimates (byse/bx):

The likelihood‐based method for uncorrelated IVs (equation (6)) is here implemented in a maximum likelihood framework, using the optim command for numerical optimization. The lack of correlation between the IV associations with the risk factor and with the outcome in the likelihood means that this code is valid in a two‐sample setting. The log‐likelihood function as defined also provides an overidentification (heterogeneity) test: under the null hypothesis that all the IVs identify the same causal effect parameter, twice the value of the log‐likelihood function at the optimum value should be distributed as a chi‐squared distribution with *K* − 1 degrees of freedom, where *K* is the total number of IVs:

The allele score estimate using summarized data for correlated IVs (equation (2)) is the same as that with uncorrelated IVs, although the standard error of the estimate (equation (8)) must be modified to account for the correlations:

The summary statistic (inverse‐variance weighted) formula valid for uncorrelated IVs can also be used with correlated IVs, although it no longer estimates a causal effect. As aforementioned, the standard error (equation (9)) must be modified to account for the correlations:

This leads to the same inferences as the aforementioned allele score estimate using summarized data for correlated IVs.

The summary statistic (inverse‐variance weighted) method for correlated IVs can be performed using weighted generalized linear regression (equations (10) and (11))

This provides an estimate of the causal effect.

The likelihood‐based method for correlated IVs (equation (A1)) is here implemented in a maximum likelihood framework, using the optim command for numerical optimization:

### 1.2 A.2 Additional tables for simulation study with uncorrelated instrumental variables

In this section, we present additional results from the simulation study with uncorrelated IVs from the main paper.

In Table A1, we report mean estimates and mean standard errors corresponding to the median estimates and median standard errors reported in Table 2. In Table A2, we report mean estimates and mean standard errors corresponding to the median estimates and median standard errors in Table 3.

**Table A1:** Comparison of allele score methods for uncorrelated instrumental variables (IVs).

	*α*	Crudely weighted	Equally weighted	Externally weighted
		Using individual‐level data		
*β*_*U*_=+1	0.05	0.342 (0.140)	0.179 (0.193)	0.172 (0.235)
	0.10	0.244 (0.081)	0.195 (0.091)	0.194 (0.093)
	0.20	0.211 (0.042)	0.199 (0.045)	0.198 (0.044)
*β*_*U*_=−1	0.05	0.056 (0.139)	0.219 (0.193)	0.230 (0.242)
	0.10	0.154 (0.081)	0.203 (0.091)	0.203 (0.092)
	0.20	0.187 (0.042)	0.200 (0.045)	0.200 (0.044)
		Using summarized data		
*β*_*U*_=+1	0.05	0.342 (0.174)	0.179 (0.214)	0.172 (0.258)
	0.10	0.244 (0.095)	0.195 (0.103)	0.194 (0.105)
	0.20	0.211 (0.049)	0.199 (0.051)	0.198 (0.050)
*β*_*U*_=−1	0.05	0.056 (0.136)	0.219 (0.179)	0.230 (0.223)
	0.10	0.154 (0.077)	0.203 (0.085)	0.203 (0.086)
	0.20	0.187 (0.040)	0.200 (0.042)	0.200 (0.041)

Mean estimates (mean standard errors) over 10000 simulations of *β*_*X*_=0.2 from simulation study with 15 uncorrelated IVs varying direction of confounding (*β*_*U*_) and average strength of IV (*α*), using allele score methods with crude weights (derived from the data under analysis), equal weights (unweighted analysis) and external weights (equivalent to estimates derived from an independent sample of equal size to the data under analysis), calculated from individual‐level and summarized data.

**Table A2:** Comparison of summarized data methods for uncorrelated instrumental variables (IVs).

	*α*	Imprecise weights	Precise weights	Oracle weights
		Allele score method using summarized data		
*β*_*U*_=+1	0.05	0.175 (0.236)	0.182 (0.204)	0.183 (0.199)
	0.10	0.197 (0.102)	0.197 (0.097)	0.197 (0.097)
	0.20	0.198 (0.049)	0.198 (0.048)	0.198 (0.048)
*β*_*U*_=−1	0.05	0.226 (0.194)	0.219 (0.168)	0.218 (0.164)
	0.10	0.205 (0.084)	0.204 (0.080)	0.204 (0.080)
	0.20	0.201 (0.040)	0.201 (0.040)	0.201 (0.040)
		Summary statistic method		
*β*_*U*_=+1	0.05	0.149 (0.166)	0.193 (0.189)	0.200 (0.192)
	0.10	0.184 (0.092)	0.199 (0.096)	0.201 (0.096)
	0.20	0.194 (0.047)	0.199 (0.048)	0.199 (0.048)
*β*_*U*_=−1	0.05	0.150 (0.136)	0.193 (0.155)	0.201 (0.158)
	0.10	0.183 (0.076)	0.199 (0.079)	0.198 (0.079)
	0.20	0.195 (0.039)	0.200 (0.040)	0.198 (0.040)
		Likelihood‐based method		
*β*_*U*_=+1	0.05	0.214 (0.212)	0.200 (0.193)	
	0.10	0.202 (0.099)	0.200 (0.096)	
	0.20	0.198 (0.049)	0.200 (0.048)	
*β*_*U*_=−1	0.05	0.213 (0.176)	0.199 (0.159)	
	0.10	0.201 (0.082)	0.200 (0.079)	
	0.20	0.199 (0.041)	0.202 (0.040)	

Mean estimates (mean standard errors) over 10000 simulations of *β*_*X*_=0.2 from simulation study with 15 uncorrelated IVs varying the direction of confounding (*β*_*U*_) and average strength of IV (*α*) using three summarized data methods: allele score, summary statistic and likelihood‐based methods, with weights taken from an external source corresponding to an independent sample of size 5000, 50000 and using the true (oracle) weights.

In Table A3, we repeated the simulations from Table 2 using *K* = 5 and *K* = 25 IVs, to increase the range of strength of the IVs considered. Results with *K* = 15 IVs are also repeated for comparison. The Monte Carlo error, representing the variability in simulation results due to the limited number of simulated datasets analysed, is 0.003 for the median estimate when *K* = 5 and *α* = 0.05 and 0.001 when *K* = 25 and *α* = 0.05. The Monte Carlo error is reduced for larger values of *α*.

**Table A3:** Further comparison of allele score methods for uncorrelated instrumental variables (IVs).

				Crudely weighted		Equally weighted		Externally weighted	
	*α*	*R* ^2^	*F*	Individual levela	Summarized data	Individual level	Summarized data	Individual level	Summarized data
*K* = 5	*β*_*U*_=+1	0.05	0.003	3.3	0.340 (0.245)	0.340 (0.305)	0.200 (0.314)	0.200 (0.362)	0.208 (0.357)	0.208 (0.415)
		0.10	0.010	10.1	0.244 (0.141)	0.244 (0.165)	0.201 (0.154)	0.201 (0.177)	0.198 (0.156)	0.198 (0.179)
		0.20	0.036	37.6	0.211 (0.073)	0.211 (0.084)	0.198 (0.077)	0.199 (0.088)	0.199 (0.075)	0.199 (0.086)
	*β*_*U*_=−1	0.05	0.003	3.3	0.065 (0.244)	0.066 (0.241)	0.205 (0.315)	0.205 (0.298)	0.201 (0.359)	0.201 (0.341)
		0.10	0.010	10.1	0.159 (0.141)	0.159 (0.134)	0.200 (0.155)	0.201 (0.145)	0.199 (0.157)	0.199 (0.147)
		0.20	0.036	37.6	0.188 (0.073)	0.188 (0.069)	0.200 (0.077)	0.200 (0.072)	0.199 (0.076)	0.199 (0.071)
*K* = 15	*β*_*U*_=+1	0.05	0.010	3.3	0.346 (0.136)	0.346 (0.171)	0.198 (0.180)	0.198 (0.204)	0.199 (0.205)	0.199 (0.235)
		0.10	0.030	10.2	0.246 (0.080)	0.246 (0.093)	0.199 (0.089)	0.199 (0.101)	0.198 (0.090)	0.198 (0.102)
		0.20	0.102	37.9	0.212 (0.042)	0.212 (0.048)	0.199 (0.044)	0.199 (0.050)	0.199 (0.043)	0.199 (0.049)
	*β*_*U*_=−1	0.05	0.010	3.3	0.052 (0.135)	0.052 (0.133)	0.201 (0.178)	0.201 (0.168)	0.200 (0.205)	0.200 (0.194)
		0.10	0.030	10.2	0.151 (0.080)	0.151 (0.076)	0.198 (0.089)	0.198 (0.083)	0.198 (0.090)	0.198 (0.084)
		0.20	0.102	37.9	0.186 (0.042)	0.186 (0.039)	0.199 (0.044)	0.199 (0.042)	0.199 (0.043)	0.199 (0.041)
*K* = 25	*β*_*U*_=+1	0.05	0.016	3.3	0.348 (0.104)	0.348 (0.131)	0.199 (0.138)	0.199 (0.157)	0.198 (0.159)	0.198 (0.181)
		0.10	0.048	10.2	0.247 (0.061)	0.247 (0.072)	0.199 (0.069)	0.199 (0.078)	0.199 (0.069)	0.199 (0.079)
		0.20	0.158	37.6	0.213 (0.033)	0.213 (0.037)	0.200 (0.034)	0.200 (0.039)	0.200 (0.033)	0.200 (0.038)
	*β*_*U*_=−1	0.05	0.016	3.3	0.051 (0.104)	0.051 (0.102)	0.201 (0.137)	0.201 (0.129)	0.200 (0.158)	0.200 (0.148)
		0.10	0.048	10.2	0.152 (0.062)	0.153 (0.058)	0.200 (0.069)	0.200 (0.064)	0.200 (0.070)	0.200 (0.065)
		0.20	0.158	37.6	0.187 (0.032)	0.188 (0.030)	0.200 (0.034)	0.201 (0.032)	0.200 (0.034)	0.200 (0.031)

Median estimates over 10000 simulations of *β*_*X*_=0.2 (median standard errors) from simulation study with uncorrelated IVs varying the number of IVs (*K* = 5,15,25), direction of confounding (*β*_*U*_) and average strength of IV (*α*; strength is also expressed by the mean values of the *R* ^2^ and *F* statistics) using allele score methods with crude weights (derived from the data under analysis), equal weights (unweighted analysis) and external weights (equivalent to estimates derived from an independent sample of equal size to the data under analysis) calculated from individual‐level and summarized data.

^a^ Median estimates and standard errors calculated from individual‐level data using a crudely weighted allele score were equal to those from a (multivariable) two‐stage least squares method to at least three decimal places in almost all simulated datasets.

The same findings were observed as in the main manuscript, with very similar estimates for allele score estimates using individual‐level and summarized data. The same pattern of median standard errors using summarized data depending on the direction of confounding was observed.

Table A4 displays the median value, coverage and power of the 95% CI for the summary statistic estimate of equation (4) calculated using crude and external weights under two values of the causal effect parameter: *β*_*X*_=0.2 (as in the simulations in the main paper), and *β*_*X*_=0 (null effect of the risk factor on the outcome). With crude weights, summary statistic estimates were biased towards the observational (confounded) association even under the null. In contrast, summary statistic estimates with external weights were biased towards the null and unbiased under the null. This means that the summary statistic method may give incorrect inference using crude weights, but using external weights will not lead to false positive conclusions because of bias from weak instruments.

**Table A4:** Investigation into bias of summary statistic estimator.

		*β*_*X*_=0.2			*β*_*X*_=0		
	*α*	Median	Coverage	Power	Median	Coverage	Power
Crude weights							
*β*_*U*_=+1	0.05	0.348	88.2	58.4	0.146	82.8	17.2
	0.10	0.248	94.5	78.2	0.047	91.7	8.3
	0.20	0.213	96.6	99.3	0.012	94.0	6.0
*β*_*U*_=−1	0.05	0.056	77.9	7.3	−0.149	83.0	17.0
	0.10	0.152	88.3	53.0	−0.047	91.9	8.1
	0.20	0.188	91.8	99.5	−0.013	94.2	5.8
External weights							
*β*_*U*_=+1	0.05	0.144	93.7	15.5	−0.001	95.2	4.8
	0.10	0.183	94.2	52.4	0.000	95.1	4.9
	0.20	0.196	94.4	97.9	0.000	94.9	5.1
*β*_*U*_=−1	0.05	0.150	92.8	19.8	0.002	94.8	5.2
	0.10	0.182	93.7	67.7	0.000	95.2	4.8
	0.20	0.196	94.1	99.7	0.000	95.2	4.8

Median estimates over 10000 simulations from summary statistic method with causal effect *β*_*X*_=0.2 and *β*_*X*_=0, coverage (%) of nominal 95% confidence interval for the causal parameter and empirical power (%) based on nominal 95% confidence interval to detect a causal effect from simulation study with 15 uncorrelated instrumental variables (IVs) varying the direction of confounding (*β*_*U*_) and average strength of IV (*α*) with crude weights and with external weights corresponding to an independent sample of size 5000.

### 1.3 A.3 Likelihood‐based method with correlated instrumental variables

The likelihood‐based method estimate with correlated IVs can be evaluated by maximizing the likelihood from the following model:

(A1) $$\begin{array}{cc} {\hat{\beta }}_{X} & ∼N_{K}\left(\xi_{k},\Sigma_{X}^{2}\right)\\ {\hat{\beta }}_{Y} & ∼N_{K}\left(\beta \xi_{k},\Sigma_{Y}^{2}\right) \end{array}$$

where $N_{K}$ indicates a *K*‐variate normal distribution. The vectors ${\hat{\beta }}_{X}=({\hat{\beta }}_{X1}\ldots {\hat{\beta }}_{\mathrm{XK}})$ and ${\hat{\beta }}_{Y}$ are of length *K* and represent the genetic associations with the risk factor and outcome respectively for IVs *k* = 1,…,*K*. The *k*_1_,*k*_2_th term of the variance–covariance matrix Σ_*X*_ is $\Sigma_{Xk_{1}k_{2}}=\sigma_{Xk_{1}}\sigma_{Xk_{2}}\rho_{k_{1}k_{2}}$, where *ρ* represents the correlation between the IVs, similarly for Σ_*Y*_. The lack of correlation between the ${\hat{\beta }}_{\mathrm{Xk}}$ and ${\hat{\beta }}_{\mathrm{Yk}}$ parameters reflects the two‐sample setting; evidence for the genetic associations with the risk factor and with the outcome is assumed to come from separate datasets (external weights). If the IV association estimates with the risk factor and outcome are obtained on the same individuals, then a 2*K*‐variate joint normal distribution can be assumed for the $\left({\hat{\beta }}_{X}{\hat{\beta }}_{Y}\right)$ parameters.

### 1.4 A.4 Additional tables for simulation study with correlated instrumental variables

In Table A5, we report mean estimates and mean standard errors corresponding to the median estimates and median standard errors reported in Table 4. In Table A6, we report mean estimates and mean standard errors corresponding to the median estimates and median standard errors reported in Table 5.

**Table A5:** Comparison of allele score methods for correlated instrumental variables (IVs).

	*α*	Allele score using individual‐level data	Allele score using summarized data
		Positive causal effect: *β*_*X*_=0.2	
*β*_*U*_=+1	0.05	0.193 (0.123)	0.193 (0.135)
	0.10	0.199 (0.060)	0.199 (0.067)
	0.20	0.200 (0.030)	0.200 (0.034)
*β*_*U*_=−1	0.05	0.211 (0.124)	0.211 (0.112)
	0.10	0.201 (0.060)	0.201 (0.055)
	0.20	0.200 (0.030)	0.201 (0.028)
		Null causal effect: *β*_*X*_=0	
*β*_*U*_=+1	0.05	−0.007 (0.123)	−0.007 (0.121)
	0.10	−0.002 (0.060)	−0.002 (0.060)
	0.20	−0.001 (0.030)	−0.001 (0.030)
*β*_*U*_=−1	0.05	0.007 (0.123)	0.007 (0.121)
	0.10	0.003 (0.060)	0.003 (0.060)
	0.20	0.001 (0.030)	0.001 (0.030)

Mean estimates (mean standard errors) over 10000 simulations of *β*_*X*_=0.2 or *β*_*X*_=0 from simulation study with 15 correlated IVs varying direction of confounding (*β*_*U*_) and average strength of IV (*α*) using allele score methods calculated from individual‐level and summarized data, with equal weights.

**Table A6:** Comparison of summarized data methods for correlated instrumental variables (IVs).

	*α*	Allele score using individual‐level data	Allele score using summarized data	Weighted generalized linear regression	Likelihood‐based method
		Positive causal effect: *β*_*X*_=0.2			
*β*_*U*_=+1	0.05	0.192 (0.130)	0.192 (0.143)	0.146 (0.112)	0.202 (0.144)
	0.10	0.199 (0.061)	0.199 (0.068)	0.184 (0.063)	0.197 (0.068)
	0.20	0.200 (0.031)	0.200 (0.034)	0.196 (0.033)	0.192 (0.033)
*β*_*U*_=−1	0.05	0.212 (0.131)	0.212 (0.118)	0.148 (0.093)	0.206 (0.122)
	0.10	0.201 (0.062)	0.201 (0.056)	0.182 (0.052)	0.196 (0.056)
	0.20	0.201 (0.031)	0.201 (0.028)	0.196 (0.027)	0.185 (0.026)
		Null causal effect: *β*_*X*_=0			
*β*_*U*_=+1	0.05	−0.007 (0.131)	−0.007 (0.128)	0.002 (0.101)	0.009 (0.125)
	0.10	−0.002 (0.062)	−0.002 (0.062)	0.000 (0.057)	0.002 (0.060)
	0.20	−0.001 (0.031)	−0.001 (0.030)	−0.001 (0.029)	0.000 (0.030)
*β*_*U*_=−1	0.05	0.008 (0.131)	0.008 (0.128)	−0.001 (0.101)	0.005 (0.126)
	0.10	0.003 (0.062)	0.003 (0.062)	0.001 (0.057)	0.003 (0.060)
	0.20	0.001 (0.030)	0.001 (0.030)	0.000 (0.029)	0.001 (0.029)

Median estimates (mean standard errors) over 10000 simulations of *β*_*X*_=0.2 or *β*_*X*_=0 from simulation study with 15 correlated IVs varying direction of confounding (*β*_*U*_) and average strength of IV (*α*), using allele score method calculated from individual‐level data, and allele score, weighted generalized linear regression and likelihood‐based methods all calculated from summarized data, with external (*N* = 5000) weights.

In Table A7, we repeated the results from Table 5 for the allele score method calculated using individual‐level data, the allele score method using summarized data, the summary statistic method calculated using weighted generalized regression and the likelihood‐based method with correlated IVs, except using crude weights generated from the data under analysis.

**Table A7:** Further comparison of summarized data methods with correlated variants.

	*α*	Allele score using individual‐level data	Allele score using summarized data	Weighted generalized linear regression	Likelihood‐based method
Positive causal effect: *β*_*X*_=0.2					
*β*_*U*_=+1	0.05	0.225 (0.112) [52.8]	0.225 (0.126) [43.7]	0.280 (0.117) [66.9]	0.308 (0.128) [69.3]
	0.10	0.208 (0.058) [89.2]	0.208 (0.065) [86.5]	0.224 (0.063) [93.0]	0.228 (0.064) [92.9]
	0.20	0.201 (0.030) [99.9]	0.201 (0.033) [99.9]	0.206 (0.032) [100.0]	0.200 (0.032) [99.9]
*β*_*U*_=−1	0.05	0.175 (0.113) [33.8]	0.175 (0.104) [41.0]	0.122 (0.097) [26.9]	0.146 (0.108) [30.5]
	0.10	0.192 (0.058) [89.3]	0.192 (0.054) [90.7]	0.177 (0.052) [88.4]	0.184 (0.054) [92.0]
	0.20	0.198 (0.030) [100.0]	0.199 (0.027) [100.0]	0.195 (0.026) [100.0]	0.184 (0.026) [100.0]
Null causal effect: *β*_*X*_=0					
*β*_*U*_=+1	0.05	0.024 (0.112) [6.1]	0.024 (0.114) [5.3]	0.079 (0.106) [11.6]	0.090 (0.115) [13.4]
	0.10	0.006 (0.059) [5.4]	0.006 (0.059) [4.9]	0.023 (0.056) [5.3]	0.023 (0.058) [6.5]
	0.20	0.002 (0.030) [5.1]	0.002 (0.030) [4.9]	0.006 (0.029) [5.3]	0.005 (0.029) [5.1]
*β*_*U*_=−1	0.05	−0.027 (0.112) [6.6]	−0.027 (0.113) [5.9]	−0.080 (0.106) [12.3]	−0.077 (0.113) [11.5]
	0.10	−0.007 (0.058) [5.4]	−0.007 (0.059) [5.1]	−0.023 (0.056) [7.0]	−0.019 (0.057) [5.9]
	0.20	−0.001 (0.030) [5.3]	0.001 (0.030) [5.1]	−0.006 (0.029) [5.6]	−0.005 (0.029) [4.8]

Median estimates over 10000 simulations of *β*_*X*_=0.2 or *β*_*X*_=0 (median standard errors) [power (%) based on nominal 95% confidence interval] from simulation study with 15 correlated instrumental variables (IVs) varying direction of confounding (*β*_*U*_) and average strength of IV (*α*) using allele score method calculated from individual‐level data and allele score, weighted generalized linear regression and likelihood‐based methods all calculated from summarized data, with crude weights.

The results show bias in the direction of the confounded observational association depending on the direction of confounding. The coverage levels under the null causal effect (the complement of the power to detect a causal effect that is expressed in Table A7) are below nominal levels, indicating that a type I error will occur under the null with greater than 5% frequency for a nominal 5% test. These phenomena are particularly marked for the summary statistic and likelihood‐based methods and less evident in the allele score methods. However, the strength of IVs considered in this example with *α* = 0.1 and *α* = 0.2 is perhaps larger than would be expected in the context of Mendelian randomization for the majority of risk factors.

### 1.5 A.5 Additional simulation study with correlated instrumental variables and binary outcomes

Following concerns from a reviewer that the simulation studies of this paper concentrated on the continuous outcome case, we repeated the simulation study of Section 4.3 (correlated genetic variants) except with a binary outcome. The data‐generating model and parameters were the same as in the main body of the paper for correlated genetic variants (equation (12)), except that the outcome model

$$y_{i}=\beta_{X}x_{i}+\beta_{U}u_{i}+\varepsilon_{\mathrm{Yi}}$$

was replaced by

$$\begin{array}{cc} \text{logit}(\pi_{i}) & =-2+\beta_{X}x_{i}+\beta_{U}u_{i}+\varepsilon_{\mathrm{Yi}}\\ y_{i} & ∼\text{Binomial}(1,\pi_{i}) \end{array}$$

The associations between IVs and the risk factor are estimated using linear regression, whereas associations between IVs and the outcome (and between the allele score and the outcome with individual‐level data) are estimated using logistic regression. Otherwise, summarized data methods proceed exactly as in the continuous outcome setting. The allele score method with individual‐level data proceeds as the ratio of the coefficient from regression of the outcome on the IV (from logistic regression) divided by the coefficient from regression of the risk factor on the IV (from linear regression). Estimates are presented using external weights only in Table A8; these results correspond to those in Table 5 of the paper.

**Table A8:** Comparison of summarized data methods for correlated instrumental variables (IVs) with binary outcome.

	*α*	Allele score using individual‐level data	Allele score using summarized data	Weighted generalized linear regression	Likelihood‐based method
Positive causal effect: *β*_*X*_=0.2					
*β*_*U*_=+1	0.05	0.150 (0.210) [10.9]	0.139 (0.191) [11.1]	0.119 (0.172) [10.8]	0.160 (0.201) [14.0]
	0.10	0.148 (0.102) [31.6]	0.147 (0.100) [32.2]	0.143 (0.095) [33.4]	0.153 (0.101) [34.8]
	0.20	0.146 (0.049) [80.6]	0.147 (0.049) [81.0]	0.147 (0.048) [83.3]	0.149 (0.049) [83.0]
*β*_*U*_=−1	0.05	0.159 (0.218) [11.7]	0.149 (0.199) [12.0]	0.131 (0.178) [11.5]	0.174 (0.209) [14.8]
	0.10	0.160 (0.106) [31.6]	0.159 (0.104) [35.4]	0.155 (0.099) [36.3]	0.165 (0.104) [37.5]
	0.20	0.158 (0.051) [80.6]	0.159 (0.051) [84.1]	0.159 (0.049) [86.3]	0.162 (0.050) [86.2]
Null causal effect: *β*_*X*_=0					
*β*_*U*_=+1	0.05	0.000 (0.219) [5.2]	0.004 (0.199) [5.2]	0.015 (0.179) [5.3]	0.018 (0.208) [6.6]
	0.10	0.000 (0.108) [5.0]	0.002 (0.106) [5.0]	0.010 (0.101) [5.4]	0.010 (0.105) [5.5]
	0.20	0.000 (0.054) [4.9]	0.002 (0.054) [4.9]	0.005 (0.052) [5.2]	0.005 (0.053) [4.8]
*β*_*U*_=−1	0.05	−0.003 (0.220) [4.6]	0.002 (0.199) [4.6]	0.013 (0.179) [4.8]	0.017 (0.208) [6.0]
	0.10	0.000 (0.108) [4.9]	0.003 (0.106) [4.8]	0.009 (0.101) [4.3]	0.009 (0.105) [5.3]
	0.20	0.000 (0.054) [4.8]	0.001 (0.054) [4.8]	0.004 (0.052) [4.8]	0.004 (0.052) [4.3]

Median estimates over 10000 simulations of *β*_*X*_=0.2 or *β*_*X*_=0 (median standard errors) [power (%) based on nominal 95% confidence interval] from simulation study with 15 correlated IVs varying direction of confounding (*β*_*U*_); and average strength of IV (*α*), using allele score method calculated from individual‐level data, and allele score, weighted generalized linear regression and likelihood‐based methods all calculated from summarized data, with external (*N* = 5000) weights.

Results are very similar with binary outcomes as with continuous outcomes. Median estimates are away from the null with a positive causal effect and close to the null with a null causal effect, and type 1 error rates are close to nominal levels under the null. Notable differences include attenuation towards the null with a positive causal effect (this is due to the non‐collapsibility of the odds ratio 22, 46), increased median standard errors and reduced power to detect a causal effect (reflecting decreased information as a result of dichotomizing the linear predictor logit(*π*_*i*_) to give a binary outcome) and limited bias under the null in the weighted generalized linear regression and likelihood‐based methods. However, the magnitude of this bias is not large, and bias did not lead to misleading inference (rejection rates were still close to the nominal 5% level). Our conclusion is that the methods presented in this paper will lead to appropriate causal inferences with binary outcomes.

### 1.6 A.6 Additional table for applied example

We provide additional information on the genetic variants used in the applied example from the main paper to estimate the causal effect of LDL‐c on CHD risk.

The genetic variants are all located in a 10‐kilobase pair region from position 55260000 to 55270000 on chromosome 1 (all positions are from build hg18). Nineteen SNPs in this region were available in both the GLGC and CARDIoGRAM datasets. Of these, two did not have information on linkage disequilibrium in the SNAP database. A further seven variants were omitted because of linkage disequilibrium with another variant at a correlation of *r* ^2^>0.8; one of each pair of correlated variants was excluded from the analysis at random in turn until no variants with a pairwise correlation above the pruning threshold remained. This was because highly correlated variants will not add to the precision of an analysis but may distort the univariate weights used in the externally weighted analyses. Variants were not included or excluded from the analysis based on their observed association with the risk factor so as to avoid bias through data‐driven selection of IVs 51.

Table A9 provides for each SNP: the rsid, position (build hg18), the coding and non‐coding alleles (in each case, the coding allele is taken as the risk factor‐increasing allele), the frequency of the coding allele taken from the CARDIoGRAM dataset, the beta‐coefficient and standard error for the genetic association with LDL‐c (the increase in standard deviation units per additional copy of the coding allele) and the beta‐coefficient and standard error for the genetic association with CHD risk (the log odds ratio per additional copy of the coding allele). The primary SNP, rs11206510, has the largest magnitude of association with the risk factor, and the IV estimate based on this SNP alone is more precise than the IV estimate based on any other single SNP.

**Table A9:** Genetic variants located in *PCSK9* gene region on chromosome 1 used in applied example from main paper: rsid, position (hg18), coding and non‐coding alleles, frequency of the coding allele, beta‐coefficient for association with LDL‐c with SE taken from GLGC, beta‐coefficient for association with CHD risk taken from CARDIoGRAM.

rsid	Position	Coding/non‐coding allele	Coding allele frequency	Association with LDL‐c (SE)	Association with CHD risk (SE)
rs1887552	55 260 222	A/T	0.29	0.037 (0.006)	0.018 (0.017)
rs11588151	55 260 236	A/G	0.81	0.059 (0.008)	0.072 (0.024)
rs9436961	55 261 419	T/A	0.27	0.046 (0.006)	0.019 (0.017)
rs2479418	55 267 465	G/A	0.49	0.018 (0.005)	0.033 (0.014)
rs2479417	55 268 332	T/C	0.35	0.017 (0.006)	0.002 (0.015)
rs2495497	55 268 583	T/C	0.12	0.035 (0.008)	0.003 (0.023)
*rs11206510*	*55 268 627*	*T/C*	*0.78*	*0.083 (0.005)*	*0.080 (0.023)*
rs17192725	55 268 719	A/G	0.07	0.048 (0.011)	0.046 (0.039)
rs17111490	55 268 764	T/C	0.07	0.002 (0.014)	−0.042 (0.043)
rs2094470	55 269 890	C/T	0.10	0.036 (0.011)	0.048 (0.028)

The primary SNP (rs11206510) is displayed in italics.

LDL‐c, low‐density lipoprotein cholesterol; SE, standard error; GLGC, Global Lipids Genetics Consortium; CHD, coronary heart disease; SNP, single nucleotide polymorphism.
